# Bumetanide for Irritability in Children With Sensory Processing Problems Across Neurodevelopmental Disorders: A Pilot Randomized Controlled Trial

**DOI:** 10.3389/fpsyt.2022.780281

**Published:** 2022-02-08

**Authors:** Dorinde M. van Andel, Jan J. Sprengers, Mandy G. Keijzer-Veen, Annelien J. A. Schulp, Marc R. Lillien, Floortje E. Scheepers, Hilgo Bruining

**Affiliations:** ^1^Department of Psychiatry, University Medical Center Utrecht Brain Centre, University Medical Center Utrecht, Utrecht, Netherlands; ^2^Department of Pediatric Nephrology, Wilhelmina Children's Hospital, University Medical Center Utrecht, Utrecht, Netherlands; ^3^N=You Neurodevelopmental Precision Center, Amsterdam Neuroscience, Amsterdam Reproduction and Development, Amsterdam UMC, Amsterdam, Netherlands

**Keywords:** ASD, ADHD, epilepsy, bumetanide, RCT, irritability, sensory processing, child

## Abstract

**Background:**

Treatment development for neurodevelopmental disorders (NDDs) such as autism spectrum disorder (ASD) and attention-deficit/hyperactivity disorder (ADHD) is impeded by heterogeneity in clinical manifestation and underlying etiologies. Symptom traits such as aberrant sensory reactivity are present across NDDs and might reflect common mechanistic pathways. Here, we test the effectiveness of repurposing a drug candidate, bumetanide, on irritable behavior in a cross-disorder neurodevelopmental cohort defined by the presence of sensory reactivity problems.

**Methods:**

Participants, aged 5–15 years and IQ ≥ 55, with ASD, ADHD, and/or epilepsy and proven aberrant sensory reactivity according to deviant Sensory Profile scores were included. Participants were randomly allocated (1:1) to bumetanide (max 1 mg twice daily) or placebo tablets for 91 days followed by a 28-day wash-out period using permuted block design and minimization. Participants, parents, healthcare providers, and outcome assessors were blinded for treatment allocation. Primary outcome was the differences in ABC-irritability at day 91. Secondary outcomes were differences in SRS-2, RBS-R, SP-NL, BRIEF parent, BRIEF teacher at D91. Differences were analyzed in a modified intention-to-treat sample with linear mixed models and side effects in the intention-to-treat population.

**Results:**

A total of 38 participants (10.1 [SD 3.1] years) were enrolled between June 2017 and June 2019 in the Netherlands. Nineteen children were allocated to bumetanide and nineteen to placebo. Five patients discontinued (*n* = 3 bumetanide). Bumetanide was superior to placebo on the ABC-irritability [mean difference (MD) −4.78, 95%CI: −8.43 to −1.13, *p* = 0.0125]. No effects were found on secondary endpoints. No wash-out effects were found. Side effects were as expected: hypokalemia (*p* = 0.046) and increased diuresis (*p* = 0.020).

**Conclusion:**

Despite the results being underpowered, this study raises important recommendations for future cross-diagnostic trial designs.

## Introduction

Neurodevelopmental disorders (NDDs) manifest in early childhood and are thought to result from atypical brain development, maturation, or function. The most common NDD classes denoted by the current Diagnostic and Statistical Manual of Mental Disorders (DSM-5) (1) are autism spectrum disorder (ASD), attention-deficit/hyperactivity disorder (ADHD), intellectual disability, and learning disorders (2). They are classified according to distinctive symptoms and behaviors, but in clinical reality show a high degree of overlap and comorbidity. Furthermore, there is shared heritability between different NDDs and causal genetic risk variants are mostly not restricted to one NDD class (3, 4). The clinical validity of NDD DSM-5 classes is further complicated due to increasing recognition of extreme variability in severity and symptomatology in clinical manifestation between individuals of the same NDD class (2).

Drug development for NDD, however, is still largely focused on DSM-5 classifications disregarding heterogeneity, which may underlie the multitude of failed trials (5). In particular in the field of ASD, diagnosis centered trials have yielded highly variable treatment responses resulting in non-significant group effects. An alternative is to stratify trial cohorts on the basis of traits that are present across different NDDs. These cross-disorder traits may reflect a degree of shared developmental trajectories and common mechanistic pathways and enhance efficacy and reduce variability in treatment response in stratified trial designs (6).

Examples of cross-disorder traits in NDD are attention problems and altered sensory reactivity or also referred to as sensory processing difficulties (SPDs). Sensory processing difficulty is a highly frequently occurring symptom in ASD, ADHD, and epilepsy (7–9) described both as behavioral over- or under-responsiveness to singular or multiple types of sensory stimuli. Sensory processing difficulty interferes with opportunities to participate in learning activities (10, 11) and significantly impair quality of life for both children and caregivers (12, 13). Sensory processing is often seen as a critical cornerstone for characterizing ASD as it is hypothesized that sensory information forms the building blocks for higher-order (social and cognitive) functioning (14). Indeed, the sensory processing domain may offer an opportunity for relatively objective measurements. An important suggested mechanism in the development and maintenance of adequate sensory processing is the regulation of the balance between excitatory and inhibitory inputs (E/I) in neuronal networks (15). A concept widely recognized in the field is that mutations in NDD genes converge on a disturbed balance between E/I in neuronal networks in the brain (16–22) that occur early (first/second trimester), or in early postnatal stages (23). Although the concept is rather generic and applied in many contexts, it is well-established that cortical networks require a finely tuned coordination of E/I for sensory information processing (24) and that changes in both directions (increasing or decreasing E/I ratio) may compromise processing and lead to NDD clinical symptoms. The E/I-balance concept is further supported by NDD mouse model studies that show E/I disturbances (25, 26) and EEG abnormalities in NDD patients that suggest E/I imbalances (27–29). Many existing compounds influence components of E/I regulation and may have purpose as rational treatments in NDD. Here, we hypothesized that stratification of SPD in NDD might be a strategy to enhance effectiveness of E/I targeting compounds.

Bumetanide is an example of an E/I influencing drug repurposing candidate for ASD. This drug has been used for decades as a diuretic drug with a mild-profile of adverse effects mostly due to its effect on fluid and electrolyte homeostasis. The rationale for selecting bumetanide, a chloride importer (NKCC1) antagonist, as a candidate treatment for ASD is to shift the polarity of GABAergic signaling through modulation of intraneuronal chloride levels. Chloride concentrations in developed neurons are maintained low after birth through inactivation of NKCC1, which shifts the polarity of GABAergic signaling from depolarizing to hyperpolarizing (30, 31). Persistent NKCC1 activity and depolarizing GABA activity has been shown in several animal models of NDD to contribute to neuronal hyperexcitability (32–36). In these models, bumetanide normalized hyper excitability and NDD-related traits (32, 35, 37–39).

These studies fueled human trials in ASD (40) and epilepsy (38). A significant effect of bumetanide on core symptoms of ASD (i.e., social behavior) was shown in three placebo-controlled trials, which used the childhood autism rating scale (CARS) as the primary outcome (41–43). A fourth trial from our research group did not find an effect on the primary outcome of the Social Responsiveness Scale but showed an improvement on a more specific core symptom scale of repetitive behavior (44). To date, no RCT has tested bumetanide in ADHD and a single study in children with epilepsy was prematurely terminated, because of possible ototoxic effects in newborns, precluding conclusions on the effect on seizures (45). Overall, most ASD bumetanide trials showed variability in treatment responses between children, most likely due to etiological heterogeneity. Given the burden of frequent blood checks needed for surveillance of diuretic effects and other potential side effects, these results warrant improved trial designs in pre-stratified NDD populations.

Cross-disorder trials face certain challenges, such as the choice of inclusion measures, concomitant medication use, and outcome selection. There are several characterization questionnaires for SPD, but we lack consensus regarding diagnostic features. For a large number of children with NDD, care as usual includes medication use to ameliorate behavioral problems. Thus, to attain a representative sample of the NDD population, it is important to allow concomitant medication use. Lastly, within NDD research, there is no gold standard for outcome measures, let alone for cross-disorder sensory reactivity outcomes. In this study we selected the aberrant behavioral scale-irritability (ABC-I) as the primary endpoint as this might overlap with behavioral sensory tolerance, is a reasonable outcome measure to detect change and is a frequently used behavioral scale in various NDD trials making it suitable for cross-disorder trial designs (46, 47). Secondary endpoints included SRS-2, RBS-R, SP-NL, BRIEF parent, and BRIEF teacher. Here, we present the results of the effectiveness of bumetanide in a pilot stratified cross-NDD RCT design.

## Materials and Methods

### Study Design and Participants

The trial was designed as multicenter, patient-randomized, double-blind placebo-controlled phase-2 superiority trial testing effectiveness of 91 days bumetanide treatment followed by a 28-day wash-out period. The trial was initiated and conducted by the UMC Utrecht in the Netherlands with Jonx Groningen as participating center. Participants were recruited through outpatient clinics and advertisement on websites of the Dutch ASD parent association (NVA), epilepsy expert association (SEIN), and the Dutch ADHD parent association (Balans). The medical ethical committee of the UMC Utrecht approved the trial protocol and the study was conducted according to the principles of the Declaration of Helsinki and Good Clinical Practice (ICH-GCP). Written informed consent was obtained from all parents or legal representatives and participants received no financial compensation. The trial was registered on 25/09/2016 with registration number 2016-002875-81. The full trial protocol is available at www.umcutrecht.nl/nl/ziekenhuis/wetenschappelijk-onderzoek/de-bascet-studie.

Children with a current ASD, ADHD (according to DSM-IV-TR or DSM-5 criteria), and/or epilepsy diagnosis, aged 5–15 years and IQ ≥ 55 were eligible as participants. Children were enrolled when a diagnosis was accompanied by altered sensory reactivity, defined as a deviant score (>1 SD deviant) on the Sensory Profile for parents or teachers (SP-NL or SP-SC) (48, 49). Use of concomitant psychoactive and antiepileptic drugs (AED) was allowed, when being taken on an unadjusted dosage at least 2 months prior to baseline measures. Exclusion criteria were renal or liver insufficiency, serious unstable illnesses (including gastroenterological, respiratory, cardiovascular, endocrinologic, immunologic, hematologic disease, dehydration or hypotension, electrolyte disturbances), treatment with NSAIDS, aminoglycosides, digitalis, antihypertensive agents, indomethacin, probenecid, acetazolamide, lithium, other diuretics, stimulants (like methylphenidate and dexamphetamine, due to it assumed diametrical effects), and drugs known to have a nephrotoxic potential. Children were allowed to receive care as usual when it was initiated minimally 2 months prior to baseline measures. Amendments to eligibility criteria were made to further include patients with ADHD and/or epilepsy besides patients with ASD with or without epilepsy. Consequently, the SP-NL was used as inclusion criterion to select patients based on sensory reactivity problems rather than diagnosis. The sensory profile was chosen since it is commonly used to detect SPD and used in several case reports and drug trials. A cut-off score of 1 SD in both directions was applied since the SP-NL detects not only sensory sensitivity.

### Randomization and Blinding

Detailed descriptions of randomization and blinding practice are described in an earlier RCT with similar study design (44). In brief, participants were randomly allocated (1:1) to receive bumetanide or placebo treatment, which was provided by Tiofarma. Sequence generation, concealment, and treatment allocation was overseen by a third-party not involved in the study (i.e., Julius Center, a consultant support agency for clinical research and trials located in the UMC Utrecht). Restricted randomization was used with permuted block design randomly varying between two, four, and six participants. Treatment allocation was done automatically using minimization with a probability of 0.75 on the participant factors active epilepsy (y/n), IQ (55–75; 76–110; >110) and study center (UMC/Jonx). Participants, parents, healthcare providers, and outcome assessors were blinded for randomization, by organizing safety checks at the pediatric nephrology department of the nearby Wilhelmina Children's Hospital.

### Procedures

The study procedures adhere to the study procedures described by Sprengers et al. (44) and to which we refer for details on the procedures. The first study visit included clinical history taking by a medical doctor or psychologist, the administration of an abbreviated WISC-III intelligence test, and medical screening by a medical doctor. Besides, study outcomes were measured at baseline (D0) and repeated after treatment (D91) and 28-day wash-out (D119), similar to previous studies (42–44).

Within 45 days of the baseline visit participants were randomized (D0) and received bumetanide or placebo tablets (0.5 mg) matched for taste, smell, and viscosity, albeit without diuretic properties. The tablets were taken orally twice-daily with minimally 6 h between the administrations (e.g., typically with breakfast and dinner). Children ≤33 kg started with halved tablets (i.e., twice-daily 0.25 mg). Children >33 kg received twice-daily 1 tablet (0.5 mg), similar to Lemonnier et al. (42) with best optimal dose-response tradeoff. When blood analysis showed no abnormalities at D7, the dosage was doubled. All participating children were supplemented with 0.5 mmol/kg potassium chloride when <30 kg, or twice-daily 8 mmol potassium chloride when ≥30 kg to avoid hypokalemia.

**Figure 1:**
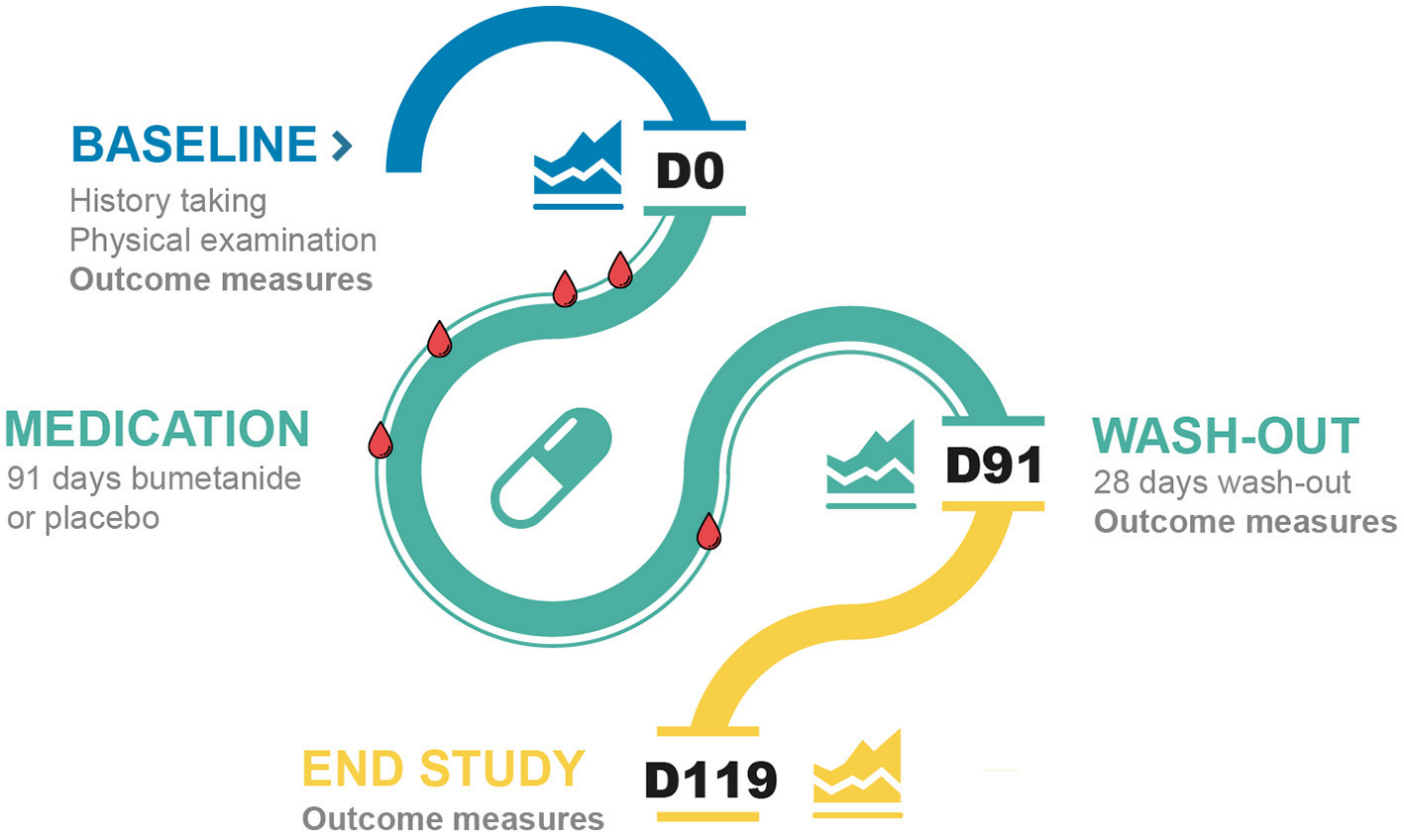
Timeline of the study visits of the trial. The blue line represents the baseline visits, the green line the medication phase and the yellow line the wash-out phase. The blood drops represent the routine blood checks. D, day.

Safety visits were scheduled at D4, D7, D14, D28, D56, D91, and D119 at the department of pediatric nephrology of the Wilhelmina Children's Hospital, with the purpose of blinding the researchers, and included blood analysis (D4, D7, D14, D28, and D56), medical evaluation, and documentation of adverse events (AEs) (adhering to NCI-CTCAE and MedDRA methodologies). Participants returned for outcome evaluations at the end of the 91-day medication phase and at the end of the 28-day wash-out period. Parents were interviewed at the last study visit (D119) about their experiences (i.e., treatment, AE, and wash-out evaluations) and were asked to predict which treatment their child had received. A schematic overview of the trial is depicted in Figure 1.

### Outcomes

The primary outcome was severity of irritable behaviors measured by the ABC-I (range 0–45; higher score is more affected) after 91 days of treatment. This measure was chosen because it is a commonly used outcome scale in (neuro)behavioral trials (50) and because we hypothesized that a beneficial clinical effect of bumetanide in this population would become evident by reducing behavioral reactivity to sensory stimuli. The SP-NL questionnaire was added to assess changes in sensory reactivity as a secondary outcome (range 125–625, lower score is more affected). However, this questionnaire was primarily developed as a screening and characterization scale and not developed to detect change. Other secondary outcomes were chosen to cover broad NDD core symptom domains; severity of restricted and repetitive behaviors, measured by the Repetitive Behavior Scale-Revised (RBS-R; range 0–129, higher score is more affected), symptom severity of social communication and social interaction, measured by the SRS-2 (range 0–195; higher score is more affected), and severity of behavioral executive functioning, measured by the Behavior Rating Inventory of Executive Function (BRIEF-parent; range 72–216; higher score is more affected) total scores at D91. To assess executive function and sensory behaviors in the school environment, the BRIEF-teacher (range 73–219; higher score is more affected) total score and domain scores of the SP-School Companion (SP-SC) were included, respectively. When participants were diagnosed with epilepsy, frequency, and type of seizures were registered with an epilepsy diary. Adverse events were passively (spontaneous report) and actively (evaluation of known side effects) collected by the nurse practitioner and physicians of the pediatric nephrology department. Incomplete individual clinical questionnaires were imputed as “no change” when less than four questions were missing (RBS-R: *n* = 1; SP-NL: *n* = 3; SP-SC: *n* = 2; BRIEF-T: *n* = 4). When four or more questions were missing, the outcome measures were excluded from analysis (*n* = 4).

### Statistical Analysis

This study was initially powered at 90% to detect an effect size of 0.5 on the primary outcome measure (ABC-I) with a standard deviation of 9.3 (i.e., mean change difference of 4.6 points), assuming two-sided alpha level of 0.05. Allowing for 10% attrition rate, 190 participants had to be randomized. Due to lower-than-expected inclusion rates, the sample size was reevaluated allowing for 80% power resulting in an intended sample size of 124 participants.

Due to the explorative nature of the study, we analyzed outcomes by modified intention-to-treat on allocated participants (see Results section for details). Screening differences between randomized and non-randomized participants were analyzed with appropriate *t*-statistics or Fisher's exact tests for dichotomized variables.

Primary and secondary outcomes at all available time points were analyzed with a linear mixed model. A random intercept was included to correct for multiple follow-up measurements per participant. Treatment and treatment by time interaction were included to assess the difference between placebo and bumetanide. In a second step, sex, age, and baseline measurement of the corresponding outcome measures were included to correct for potential confounding and optimize the statistical analysis for power (51, 52). Statistical assumptions of the models (i.e., distributional assumptions, homoscedasticity) were assessed by examining residuals (53). From these models, we derived estimated means for each treatment arm as well as a mean difference (MD) between treatment groups at 91 days with 95% confidence intervals (CI) and *p*-values. Additional analyses were performed for treatment interactions with sex, age, and total IQ and were evaluated with likelihood ratio tests (LRT). Safety was analyzed in all allocated subjects (i.e., ITT) with Fisher's exacts tests. Agreement of predictions by parents of the allocated treatment arm vs. the actual treatment allocated to children was analyzed with Cohen's kappa. All analyses were performed with SPSS v25 (IBM, Corp., Armonk, NY) and SAS v9.4 (SAS, Cary, NC).

Study safety was overseen twice a year by the Data Safety Monitoring Board (DSMB) of the UMC Utrecht. This study was registered with the EudraCT trial registry (2016-002875-81) and Dutch trial registry (NL6178).

## Results

### Participant Characteristics

Participants were enrolled between June 20th 2017 and June 26th 2019, the end of planned recruitment and funding. The study was finished without meeting the intended study population (*n* = 124), since inclusion rates were not met. Based on the initial power calculation the actual power of the study reached 27%. Multiple attempts were undertaken to increase recruitment, including adding research staff, advertisements on Dutch ASD, ADHD, and epilepsy parent associations and presentations during their meetings. However, various strategies to increase inclusions failed, which rendered extension of the trial to meet the sample size not feasible. The participating center in Groningen was unable to follow the study procedures and the few participants randomized at this site (*n* = 5) could not be included for analysis in the study due to incomplete questionnaires. No serious AEs were reported in this participating center. As a consequence, the study is reported as a single center trial.

**Figure 2:**
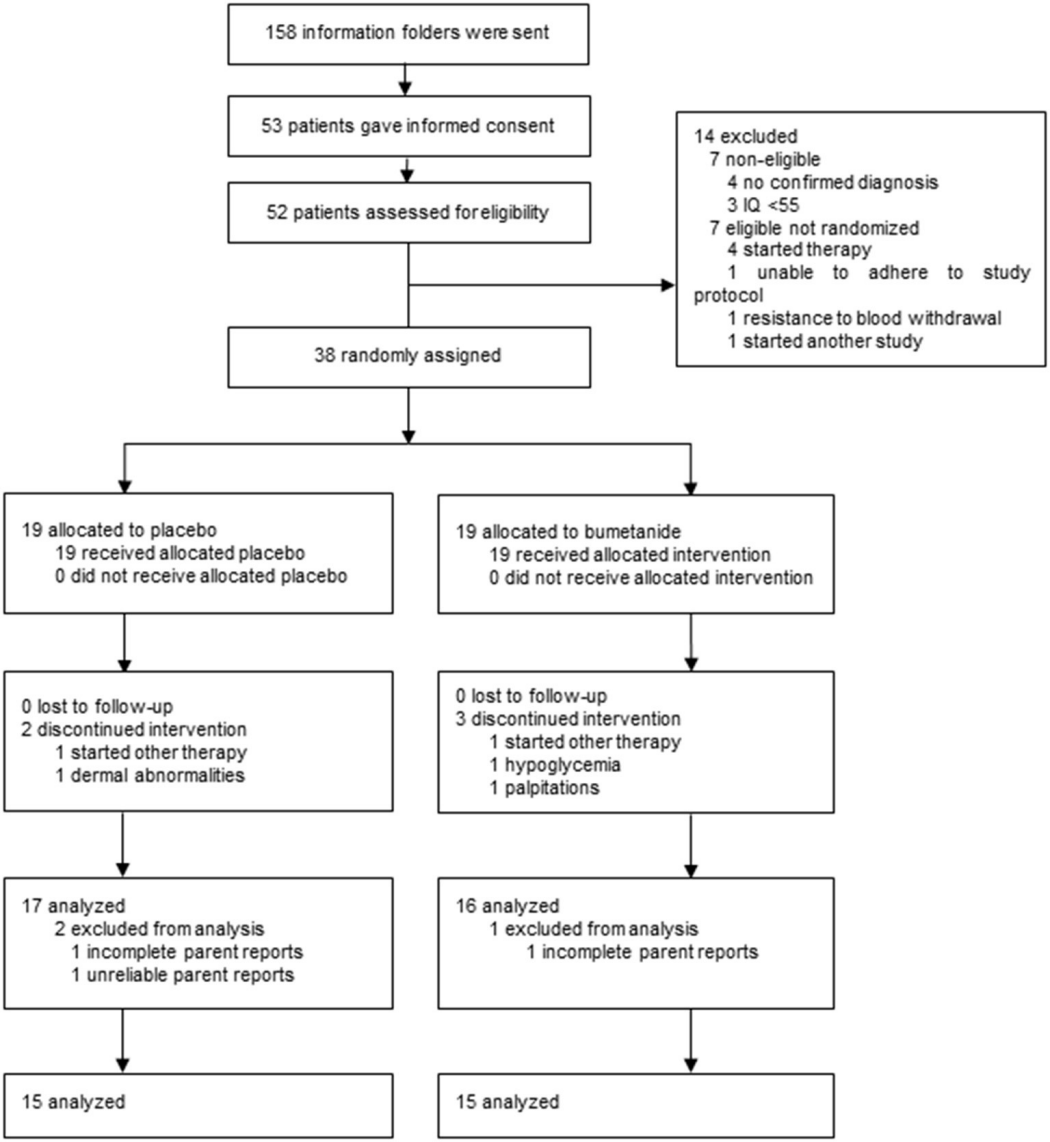
CONSORT diagram of the trial.

As shown in the CONSORT diagram in Figure 2, a total of 158 caregivers contacted the research team and obtained a study information folder. After information was sent, 53 potential participants gave informed consent and 52 were assessed for eligibility. Fourteen participants did not progress to randomization for reasons of non-eligibility (*n* = 7), requirement of immediate other therapy (*n* = 4), inability to adhere to study protocol (*n* = 1), resistance to blood withdrawal (*n* = 1), and participation in another study (*n* = 1), resulting in 38 participants that were randomly allocated to bumetanide or placebo treatment (Supplementary Table 1). There was no difference in baseline characteristics and outcomes between eligible participants who did and who did not advance to randomization (*p* ≥ 0.153).

Of the 38 randomized participants, 19 (4 female participants) were allocated to the bumetanide arm and 19 (6 female participants) to the placebo arm. Five participants discontinued treatment prior to collecting outcomes. Two were allocated to placebo: one required immediate psychiatric intervention with other therapy and one withdrew consent because of the high burden, absence of benefits, and perseverance of mild potential side effects (i.e., dermal abnormalities). The other three participants that discontinued treatment had been allocated to bumetanide: one required immediate psychiatric intervention with other therapy, one due to repeating hypoglycemia, and one due to palpitations. During the trial, none of the participants, parents, healthcare providers, or outcome assessors were unblinded. On completion of the trial and before unblinding, one participant was excluded from analyses as questionnaires were not reliable (i.e., were filled out by a different parent; placebo) and for two participants multiple D91 questionnaires were missing (1 placebo, 1 bumetanide).

Table 1 depicts baseline characteristics of the analyzed sample including (previous) medication use and diagnoses.

**Table 1:** Baseline characteristics of the analyzed population.

	**Placebo group** ***(n*** **=** **15)**		**Bumetanide group** ***(n*** **=** **15)**		**Total** ***(n*** **=** **30)**	
Age (years, SD)	8.7 (3.1)		10.9 (2.5)		9.8 (3.0)	
Sex (%) Male	10 (66.7)		12 (80.0)		22 (73.3)	
Female	5 (33.3)		3 (20.0)		8 (26.7)	
IQ (SD)	99.5 (25.3)		98.9 (24.0)		99.2 (24.2)	
**Medication use (%)**	**Prior**	**During trial**	**Prior**	**During trial**	**Prior**	**During trial**
None	7 (46.7)	11 (73.3)	9 (60.0)	12 (80.0)	16 (53.3)	23 (76.7)
AP	1 (6.7)	1 (6.7)	1 (6.7)	2 (13.3)	2 (6.7)	3 (10.0)
AED	5 (33.3)	2 (13.3)	1 (6.7)	0 (0)	6 (20.0)	2 (6.7)
SSRI	0 (0)	0 (0)	1 (6.7)	1 (6.7)	1 (3.3)	1 (3.3)
AP + benzo	0 (0)	1 (6.7)	0 (0)	0 (0)	0 (0)	1 (3.3)
Benzo	0 (0)	0 (0)	1 (6.7)	0 (0)	1 (3.3)	0 (0)
Stimulant	3 (20.0)	0 (0)	4 (26.7)	0 (0)	7 (23.3)	0 (0)
Alpha2	2 (13.3)	0 (0)	0 (0)	0 (0)	2 (6.7)	0 (0)
**Diagnoses (%)**			
ASD	11 (73.3)		11 (73.3)		22 (73.3)	
ASD only	7 (46.7)		8 (53.3)		15 (50.0)	
ASD + ADHD	3 (20.0)		3 (20.0)		6 (20.0)	
ASD + epilepsy	1 (6.7)		0 (0)		1 (3.3)	
ADHD	2 (13.3)		4 (26.7)		6 (20.0)	
Epilepsy	2 (13.3)		0 (0)		2 (6.7)	

*Data are mean (SD) or N (%). ADHD, attention deficit hyperactivity disorder; AED, antiepileptic drug; AP, antipsychotics; ASD, autism spectrum disorder; Benzo, benzodiazepine; Prior, medication history up to 8 weeks before trial start; SSRI, selective serotonin reuptake inhibitor; Y, years*.

Of all analyzed participants, 22 (73.3%) were classified with ASD with or without comorbidities, 6 (20%) with ADHD, and two (6.7%) had epilepsy (Table 1). A total of 16 (53.3%) participants were naïve for the use of psychoactive medication. At the start of (and during) the trial three participants (10%) were taking antipsychotics, two (6.7%) were taking AEDs, one participant used a selective serotonin reuptake inhibitor (SSRI) (3.3%), and one antipsychotics together with benzodiazepines. Twenty-three (76.7%) were not taking any medication.

Medication adherence was monitored via several approaches: interview, inspection of returned medication packages, and a drug diary. We found no evidence of non-adherence in either treatment group. The mean provided bumetanide dosage was 0.0430 mg/kg/day (range: 0.0182–0.0637). Treatment dose was increased at D7 in all but two participants (due to hypokalemia and a postponed safety visit and which were both increased at D14). In three participants, the target dose had to be temporarily halved for 11, 12, and 13 days, respectively, due to hypokalemia (*n* = 3, bumetanide).

We documented parent predictions of the treatment their child had received once the last study visit for the participant was completed. In the bumetanide group (*n* = 15), 12 parents expected allocation to bumetanide and three parents expected allocation to placebo. In the placebo group (*n* = 15), one parent expected allocation to bumetanide, 14 parents expected allocation to placebo, and one parent was not assessed. A substantial accordance between expected and actual treatment allocation was found (κ = 0.737 [with 0 indicative of effective blinding and 1 indicative of a potential failure of blinding], *p* = 0.000).

### Outcomes

**Figure 3:**
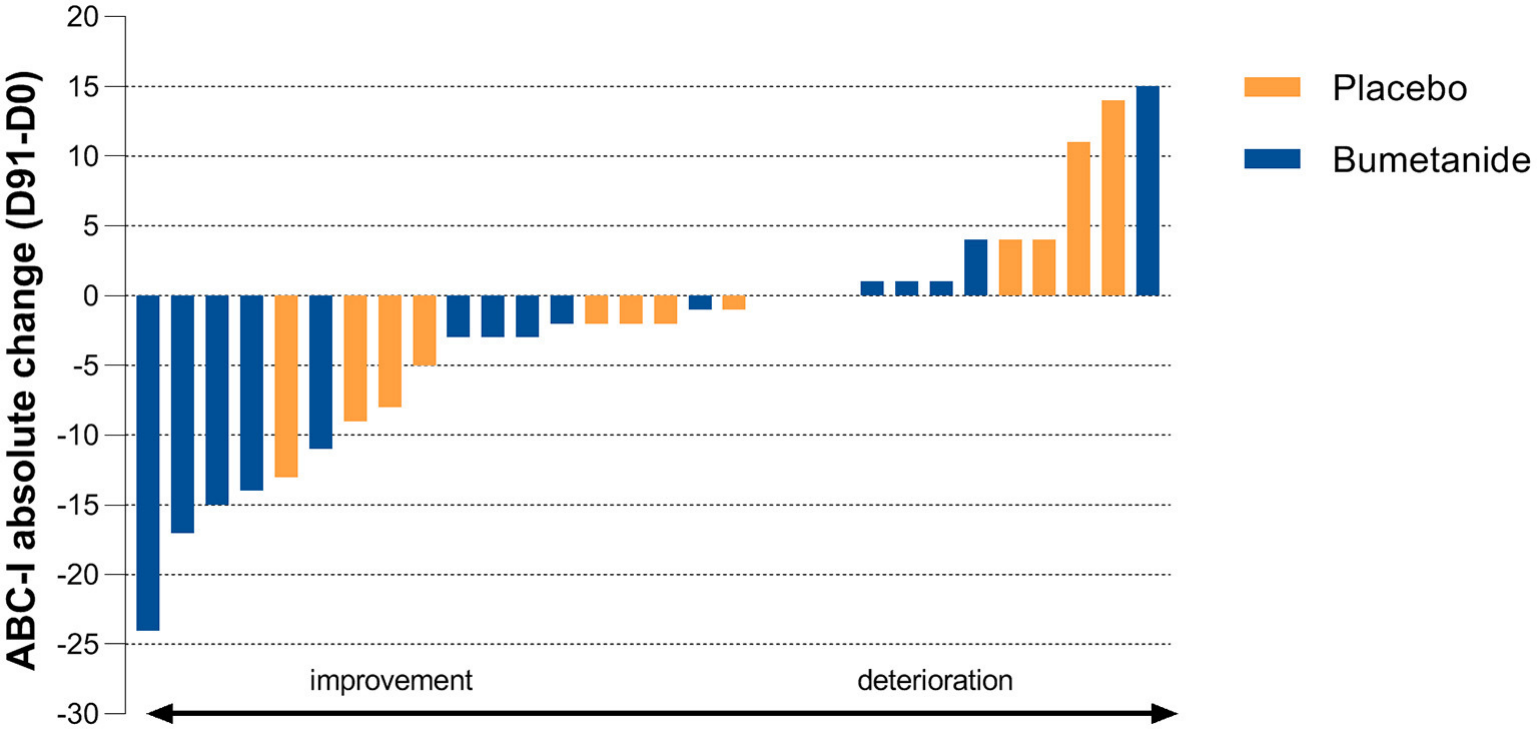
Individual treatment effect on the primary outcome. Absolute change on the Aberrant Behavior Checklist-Irritability subscale (ABC-I) after 91 days of treatment (D91 minus D0). Blue bars indicate bumetanide treatment, orange bars indicate placebo treatment. Each bar (blue or orange) represents the outcome for an individual enrolled in the trial. The primary endpoint shows a significant treatment effect (*p* = 0.0125) favoring the bumetanide group.

**Figure 4:**
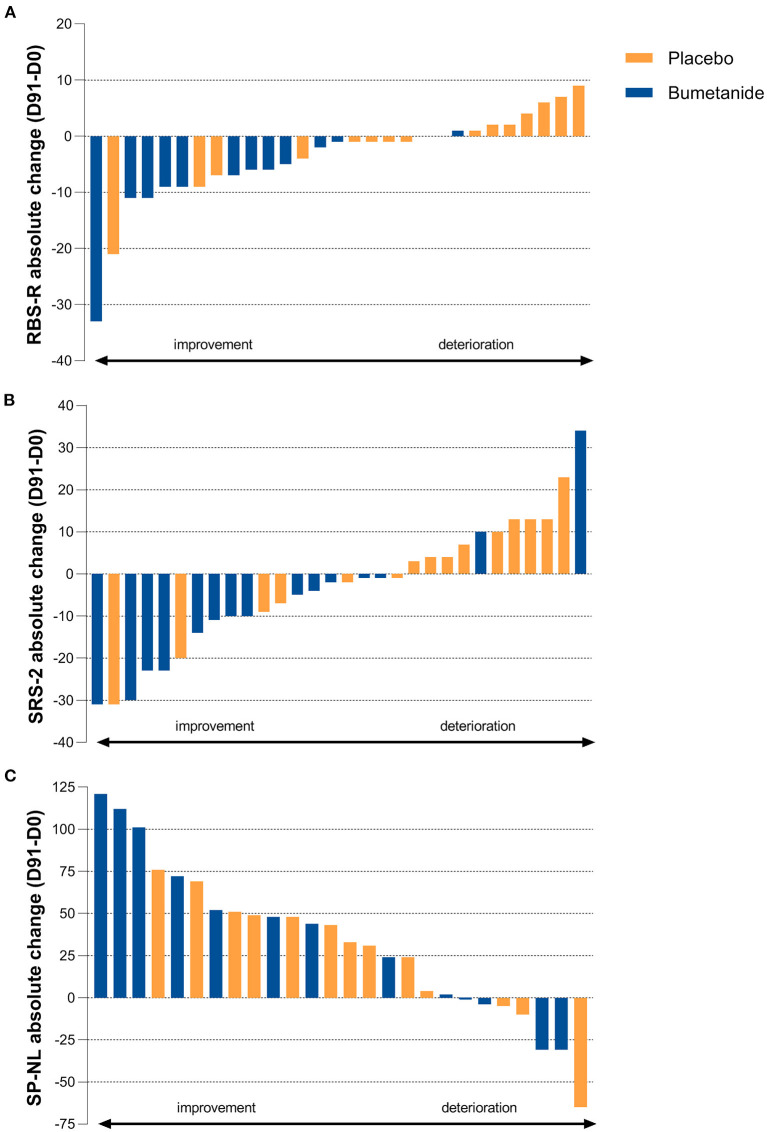
Individual treatment effect on secondary outcomes. **(A)** Absolute change on the Repetitive Behavior Checklist-Revised (RBS-R) after 91 days of treatment showing no superior treatment effect (*p* = 0.109). Blue bars indicate bumetanide treatment, orange bars indicate placebo treatment. Each bar (blue or orange) represents the outcome for an individual enrolled in the trial. **(B)** Absolute change on the Social Responsiveness Scale-2 (SRS-2) total score showing no superior treatment effect (*p* = 0.181). **(C)** Absolute change on the Sensory Profile-NL (SP-NL) total score showing no superior treatment effect (*p* = 0.844).

Bumetanide showed a superior treatment effect on severity of irritability symptoms, the primary outcome of this study (ABC-I MD: −4.78, 95%CI: −8.43 to −1.13, *p* = 0.0125; Figure 3; Table 2). No effects were found on the secondary outcomes. There was no superior effect of bumetanide on core ASD symptomatology measured with RBS-R (model adjusted for heteroscedasticity, MD: −4.90, 95%CI: −10.97 to 1.17, *p* = 0.109) and SRS-2 (MD: −6.61, 95%CI: −16.51 to 3.28, *p* = 0.181), indicating no effect of bumetanide on repetitive behaviors and social communication and social interaction (Figure 4; Table 2). Moreover, no superior effects were found on sensory symptoms measured with the SP-NL (MD: 3.14, 95%CI: −29.3 to 35.6, *p* = 0.844) nor on executive behavior (BRIEF) measured by parents (MD: −7.88, 95%CI: −17.6 to 1.8, *p* = 0.105) or teachers (MD: −3.08, 95%CI: −19.7 to 13.5, *p* = 0.698). Analyses showed no wash-out effects on any outcome. Descriptive results of the subscales are presented in Supplementary Table 2 as the study was not sufficiently powered to statistically test subscales.

**Table 2:** Changes in primary and secondary outcome measures after treatment and wash-out.

	**Placebo group**			**Bumetanide group**			**Treatment effect**	***p*-value**
	**Baseline**	**D91**	**D119**	**Baseline**	**D91**	**D119**		
**ABC-I subscale**							
*n*	15	15	11	14	14	14		
Mean	17.1 (9.1)	16.5 (8.6)	13.2 (8.8)	13.1 (10.3)	6.9 (4.3)	7.9 (6.7)	−4.78 (−8.4 to −1.1)	0.0125
**SRS-2 total**							
*n*	15	15	11	15	15	15		
Mean	80.0 (32.9)	81.3 (33.1)	88.5 (25.5)	78.9 (26.1)	70.9 (23.8)	73.9 (24.3)	−6.61 (−16.5 to 3.3)	0.181
**RBS-R total**							
*n*	15	15	11	14	14	14		
Mean	17.8 (13.0)	16.9 (12.5)	19.4 (18.0)	17.4 (16.6)	10.4 (9.4)	15.3 (14.1)	−4.90 (−11.0 to 1.2)	0.109
**SP-NL total**							
*n*	14	14	10	15	15	15		
Mean	452.7 (55.9)	477.8 (66.3)	473.8 (64.6)	442.0 (55.7)	482.3 (47.1)	472.8 (59.6)	3.14 (−29.3 to 35.6)	0.844
**BRIEF-parent total**					
*n*	15	15	11	15	15	14		
Mean	164.3 (20.0)	162.2 (19.3)	160.1 (20.7)	159.5 (21.5)	150.9 (20.0)	153.8 (17.1)	−7.88 (−17.6 to 1.8)	0.105
**BRIEF-teacher total**						
*n*	13	13	8	11	11	9		
Mean	148.8 (23.3)	145.7 (25.8)	149.0 (19.2)	148.6 (17.2)	143.3 (27.8)	141.9 (31.7)	−3.08 (−19.7 to 13.5)	0.698

*Data are means (SD). Data is shown for those participants that completed D91. Treatment effects are measured with linear mixed models (including age, gender, and baseline measurements) and shown with (95% CI). ABC-I, aberrant Behavior Checklist-Irritability (range 0–45; higher score is more affected); BRIEF, Behavior Rating Inventory of Executive Function (Parent range 72–216 and Teacher range 73–219; higher score is more affected); RBS-R, Repetitive Behaviors Scale-Revised (range 0–129; higher score is more affected). SP-NL, Sensory Profile-Dutch version (range 125–625; lower score is more affected); SRS-2, Social Responsiveness Scale-2 (range 0–195; higher score is more affected). Significance level is p < 0.05*.

Sub-analyses on treatment-by-sex, age and IQ interaction showed only a significant treatment-by-IQ interaction effect on the BRIEF-teacher (MD: −0.65, 95%CI: −1.33 to 0.02, LRT = 3.9, *p* = 0.0483), indicating that within the bumetanide group, a higher IQ was associated with higher scores after treatment, whereas in the placebo group a higher IQ was associated with lower scores.

Mean treatment dose showed no association with change in ABC-I in the bumetanide group (ρ = 0.157, *p* = 0.591) i.e., there was no dose-response relationship. Lastly, change in ABC-I showed no association with baseline SP-NL total scores in the bumetanide group (respectively, *r* = 0.438; *p* = 0.117).

### Tolerability and Adverse Effects

Adverse events that occurred in more than 5% of participants are shown in Table 3. All events were mild to moderate according to CTCAE rating-scale and all resolved. Three serious AEs occurred in two patients (both bumetanide group): anaphylactic reaction to incidental cow milk ingestion in a child with preexisting cow milk allergy, blood loss after elective adenoidectomy/tonsillectomy requiring prolonged hospital observation; and exaggeration of preexisting palpitations by sinus tachycardia. Cardiac evaluations showed no abnormalities. The serious AEs were registered as probably unrelated to study treatment, except for palpitations, which was possibly related due to hypovolemia, although no signs of hypovolemia were found on echocardiography. Common cold, myalgia, orthostatic hypotension, and hypokalemia were the most frequently occurring AEs. A total of 32% of participants in the bumetanide group experienced increased diuresis compared to none in the placebo group (*p* = 0.020). In addition, 26% of participants in the bumetanide group developed hypokalemia against none in the placebo group (*p* = 0.046). Hypokalemia occurred in one patient at D10. In the other four patients hypokalemia occurred only after D14 and potassium levels did not drop below 3.0 mmol/L and normalized with increased oral potassium chloride (Supplementary Table 3).

**Table 3:** Adverse events occurring in >5% of participants classified in MedDRA categories.

		**Bumetanide group (*****n*** **=** **19)**				**Placebo group (*****n*** **=** **19)**			
	**Symptom**	**# of AEs**	**# of part**.	**Severity**	**IR^a^**	**# of AEs**	**# of part**.	**Severity**	***p*-value**
Total AE	100	19			61	17		
Metabolism and nutrition disorders								
	Hypokalemia	9	5	Moderate	1				0.0463
	Dehydration	3	3	Moderate	1				0.230
	Hypoglycemia	2	1	Mild	3				1.000
	Hyponatremia	2	1	Moderate	2				1.000
Gastrointestinal disorders									
	Vomiting	3	3	Mild	2	3	3	Mild	1.000
	Nausea	7	6	Mild	2	3	2	Mild	0.232
	Abdominal pain	7	6	Mild	3	3	3	Mild	0.447
	Obstipation					2	2	Moderate	0.487
	Gastroenteritis	3	3	Mild	3	4	4	Mild	1.000
Vascular disorder									
	Orthostatic hypotension	9	8	Mild	1	3	3	Mild	0.151
Infections and infestations								
	Common cold	3	3	Mild	3	14	10	Mild	0.0382
Musculoskeletal and connective tissue disorders									
	Myalgia	10	7	Mild	2	3	3	Mild	0.269
	Muscle cramp	2	2	Mild	2	2	1	Mild	1.000
Renal and urinary disorders									
	Dysuria	2	2	Mild	2				1.000
	Enuresis^b^	1	1	Mild	1				1.000
	Increased diuresis	6	6	Mild	1				0.0197
Nervous system disorders									
	Headache	6	4	Mild	3	7	7	Moderate	0.476
	Dizziness	2	2	Mild	3	2	2	Mild	1.000
Psychiatric disorders									
	Insomnia	1	1	Mild	3	4	4	Mild	0.340
General disorders and administration site conditions								
	Fatigue	4	3	Mild	2	2	2	Mild	1.000
Skin and subcutaneous tissue disorders								
	Dermal abnormalities	3	3	Moderate	3	2	2	Moderate	1.000
Injury, poisoning and procedural complications					
	Injury	4	4	Moderate	3				0.105

*Data are n. Differences were tested with Fisher Exact tests. #, number; AE, adverse event; IR, intervention relationship; Part, participants*.

a *1, definitely related; 2, possibly related; 3, not related*.

b *Occurring in <5% of participants, but listed as important expected AE. Significance level is p < 0.05*.

## Discussion

Hallmarks of NDD diagnoses are etiological and clinical heterogeneity and effectiveness of trials might be augmented in cohorts ascertained by traits that may reflect an enhanced degree of shared pathophysiology. In this context, we hypothesized that SPD marks an important cross-disorder trait and tested whether bumetanide may improve sensory induced irritable behavior. Albeit the limited sample size, we found a superior effect of bumetanide on this primary endpoint. Bumetanide was well-tolerated with only mild to moderate, expected (i.e., hypokalemia and diuresis) and reversible side effects.

The observed treatment effect in this pre-stratified sample is encouraging. Existing treatment options to reduce irritable behavior in NDDs are limited to antipsychotics such as risperidone and aripiprazole that may owe their effect to sedative, symptomatic properties with detrimental side effects. Bumetanide is an attractive alternative due to its rational mechanism suggested from a large body of experimental research (35).

We encountered several challenges during this pilot trial that may be improved when future studies consider a similar design. We expected that a trial design with recruitment based on traits would be more appealing for participants than trials following classical inclusion based upon ASD and ADHD diagnoses. Hence, the inclusion difficulties were contrary to our expectations. The study failed (*n* = 38) to meet the recruitment target (*n* = 124), resulting in a power of 27% which is undesirable but not uncommon: a review estimated the median achieved power of studies in neurosciences between 8 and 31% (54). Several aspects seem to have contributed to problematic recruitment. First, healthcare support, access to special education, and referral systems in the Netherlands are still organized along DSM-classifications, which might render certain patients and caregivers reluctant to participate. Second, the placebo-controlled trial design was frequently mentioned as reason to decline participation. Some children requiring drug intervention were expected not to endure a period of placebo allocation. Indeed, caregivers of children on psychostimulants, an exclusion criterion due to its expected diametrical effect to bumetanide, were eager to participate due to experienced side effects (e.g., rebound, sleeping problems, and emotional blunting). For them, bumetanide was appealing as a safe alternative, although they were hesitant to stop medication for the duration of the trial and risk deterioration in school performance and family stability. Third, we suspect that the limited participation of children with epilepsy was due to the treatment focus on seizure management instead of behavioral problems by parents, pediatricians, and neurologists. Taken together, it seems that several reasons may have hampered the readiness for cross-disorder trait approaches, which may be improved by including for instance comparative trial designs.

Another evident challenge is the development of more appropriate clinical outcomes. Although there are excellent assessment tools to characterize SPD, the most prominent being the Sensory Profile-NL these have limited applicability to detect treatment effect. As a consequence, the assumption that improvements in irritable behavior are mediated by improvements in sensory behavior could not be tested. No correlation was found between change in ABC-I and baseline SP-NL, however, this might have been influenced by insufficient power or psychometrically inadequate properties of the SP-NL total score in which (clinically) more severely affected children do not necessarily show highest SP-NL scores. For now, the ABC-I, however, is a reasonable outcome measure to detect change and is a frequently used behavioral scale in NDD trials (46, 47). Still, there is a great need for suitable outcomes that can more directly measure certain expected mechanistic effects, preferably scales that can be individually adapted (55) as treatment response variability between subjects varies greatly in trials. Future trials may benefit by personalizing instead of specifying clinical outcome measures. In this way, improvement in debilitating behaviors can be evaluated which results in more notable and valuable improvements in daily life. The inclusion of diagnostic companions (e.g., electroencephalography) to bridge clinical to assumed mechanistic effects offers another opportunity. In this trial, it may have demonstrated mechanistic insights on central nervous system effects given the limited brain availability of bumetanide (38).

In addition to these challenges we highlight several limitations that obstruct interpretation of our findings. An underpowered study is problematic as it reduces the chance of detecting true effects (in outcomes but also in AEs) and also reduces the likelihood that the significant treatment effect on irritable behavior reflects a true, replicable effect. The small sample size did not allow for subgroup analyses precluding recommendations for specific NDD classifications. Further, as all participants with epilepsy were allocated to placebo, we have no record of the potential effect of bumetanide on seizure frequency. To improve this in future trials it would be recommended to perform trials across diagnosis specific expertise centers in order to balance recruitment per diagnosis.

Another limitation is that functional unblinding may have interfered with the results. There was substantial agreement between expected and actual treatment allocation as reported by parents. In addition, expected diuretic side effects were restricted to the bumetanide group. While several parents (7 out of 14) claim that their prediction was based on clinical improvement, we should adopt a more conservative interpretation as adverse effects may have contributed to unblinding. Indeed, diuretic side effects (i.e., increased diuresis, hypokalemia, enuresis, and dysuria) occurred in 10 out of 19 participants treated with bumetanide. Functional unblinding is a concern in bumetanide RCTs due to its renal effects. To prevent unblinding, we followed the same rigorous procedures that were used in our previous bumetanide RCT in ASD, where no indication of insufficient blinding was found (44). All participants started with potassium chloride supplementation and the researchers were blinded for safety controls and side effects. Despite our rigorous efforts, adequate blinding remains a challenge in bumetanide RCTs that not include comparatives with diuretic properties.

Fortunately, there is a growing awareness to develop new trial approaches moving from traditional medicine to personalized or stratified approaches, such as the Research Domain Criteria initiative (RdoC) (56). A primary assumption of RDoC is that interventions are more effective if heterogeneity within and amongst disorders is reduced, for instance by symptom stratification. Future trial designs may adopt these approaches and start to move away from one-size-fits-all to more stratified therapy and increase the benefit-risk ratio for patients.

Here, we have presented a pilot RCT based on a stratified trial design in which we found a superior effect (modified ITT analysis) of bumetanide on irritable behavior in children with NDD and SPD. Although the small sample size and potential functional unblinding do not allow firm conclusions or generalizability of treatment effect, these results encourage future studies that implement SPD stratification in testing bumetanide or similar agents. Our recommendations for future trials include dedicated expertise centers for balanced recruitment across diagnoses, consideration of using comparatives with diuretic properties to reduce the risk of functional unblinding (in the specific case of bumetanide) and improvement of cross-disorder inclusion and outcome measures.

## Data Availability Statement

The original contributions presented in the study are included in the article/Supplementary Materials, further inquiries can be directed to the corresponding author/s.

## Ethics Statement

The studies involving human participants were reviewed and approved by Medical Ethical Committee of the UMC Utrecht. Written informed consent to participate in this study was provided by the participants' legal guardian/next of kin.

## Author Contributions

HB designed the study. DA and JS contributed to acquisition and analysis of the data. MK-V, ML, and AS collected data on side effects and compliance. DA, JS, and HB interpreted the patient data and wrote the initial manuscript. All authors contributed to paper revisions and approved the final manuscript.

## Funding

This study was supported by a grant from Dutch Brain Foundation (Hersenstichting #HA2015-01-04). This foundation had no role in the design of the study and collection, analysis, and interpretation of data and in writing the manuscript.

## Conflict of Interest

HB has reported being a shareholder of Aspect Neuroprofiles BV, which provides EEG-analysis services for clinical trials. The remaining authors declare that the research was conducted in the absence of any commercial or financial relationships that could be construed as a potential conflict of interest.

## Publisher's Note

All claims expressed in this article are solely those of the authors and do not necessarily represent those of their affiliated organizations, or those of the publisher, the editors and the reviewers. Any product that may be evaluated in this article, or claim that may be made by its manufacturer, is not guaranteed or endorsed by the publisher.
