# Supplementary material for: Bumetanide for Irritability in Children With Sensory Processing Problems Across Neurodevelopmental Disorders: A Pilot Randomized Controlled Trial
Source: Front Psychiatry. 2022 Feb 8;13:780281. doi: 10.3389/fpsyt.2022.780281 (PMC8861379; doi:10.3389/fpsyt.2022.780281)
Supplement: Supplementary file 1 [file Data_Sheet_1.docx]

Supplementary Material

# Supplementary table 1

| **Table S1.** Baseline characteristics of the intention to treat population | | | | | | | | |
| --- | --- | --- | --- | --- | --- | --- | --- | --- |
|  | | | **Placebo group (n=19)** | | **Bumetanide group (n=19)** | | **Total (n=38)** | |
| **Age (y, SD)** | | | 9.0 (3.3) | | 11.2 (2.6) | | 10.1 (3.1) | |
| **Sex (%)** | | Male | 13 (68) | | 15 (79) | | 28 (74) | |
|  | | Female | 6 (32) | | 4 (21) | | 10 (26) | |
| **IQ (SD)** | | | 98.3 (22.6) | | 97.1 (22.9) | | 97.7 (22.4) | |
| **Medication use (%)** | | | **Prior** | **During trial** | **Prior** | **During trial** | **Prior** | **During trial** |
| None | | | 9 (47.4) | 13 (68.4) | 9 (47.4) | 13 (68.4) | 18 (47.4) | 26 (68.4) |
| AP | | | 2 (10.5) | 1 (5.3) | 4 (21.1) | 3 (15.8) | 6 (15.8) | 4 (10.5) |
| AP + benzo | | | 0 (0) | 1 (5.3) | 0 (0) | 0 (0) | 0 (0) | 1 (2.6) |
| Benzo | | | 1 (5.3) | 0 (0) | 1 (5.3) | 0 (0) | 2 (5.3) | 0 (0) |
| AED | | | 6 (31.6) | 3 (15.8) | 1 (5.3) | 0 (0) | 7 (18.4) | 3 (7.9) |
| SSRI | | | 1 (5.3) | 0 (0) | 2 (10.5) | 2 (10.5) | 3 (7.9) | 2 (5.3) |
| SSRI + AP | | | 0 (0) | 1 (5.3) | 0 (0) | 1 (5.3) | 0 (0) | 2 (5.3) |
| Stimulant | | | 5 (26.3) | 0 (0) | 6 (31.6) | (0) | 11 (28.9) | (0) |
| Alpha2 | | | 2 (10.5) | 0 (0) | 1 (5.3) | (0) | 3 (7.9) | (0) |
| **Diagnoses (%)** | | |  | |  | |  | |
| ASD | | | 15 (78.9) | | 15 (78.9) | | 30 | |
|  | ASD only | | 10 (52.6) | | 12 (63.2) | | 22 (57.9) | |
|  | ASD + ADHD | | 4 (21.1) | | 3 (15.8) | | 7(18.4) | |
|  | ASD + epilepsy | | 1 (5.3) | | 0 (0) | | 1 (2.6) | |
| ADHD | | | 2 (10.5) | | 4 (21.1) | | 6 (15.8) | |
| Epilepsy | | | 2 (10.5) | | 0 (0) | | 2 (5.3) | |
| **Note:** Data are mean (SD) or N (%). ADHD = attention deficit hyperactivity disorder; AED = antiepileptic drug; AP = antipsychotics; ASD = autism spectrum disorder; Benzo = benzodiazepine; Prior = medication history up to 8 weeks before trial start; SSRI = selective serotonin reuptake inhibitor; Y = years. | | | | | | | | |

# Supplementary table 2

**Table S2.** Mean and standard deviations of subscales at different time points

|  | **Placebo group** | | | | **Bumetanide group** | | |
| --- | --- | --- | --- | --- | --- | --- | --- |
|  | **Baseline** | **D91** | **D119** | **Baseline** | | **D91** | **D119** |
| **SRS-2 subscales** | n=15 | n=15 | n=11 | n=15 | | n=15 | n=15 |
| Social Awareness | 10.9 (4.5) | 11.6 (4.5) | 12.6 (3.4) | 10.9 (4.0) | | 9.9 (3.9) | 9.9 (3.5) |
| Social Cognition | 15.6 (6.2) | 15.1 (8.0) | 16.0 (4.9) | 14.2 (5.5) | | 13.9 (4.9) | 14.7 (5.5) |
| Social Communication | 26.8 (11.4) | 26.2 (9.8) | 28.5 (8.7) | 25.7 (8.9) | | 22.9 (8.7) | 23.7 (8.6) |
| Social Motivation | 14.1 (7.6) | 15.1 (6.3) | 15.6 (6.7) | 13.9 (5.2) | | 12.3 (4.6) | 12.7 (5.5) |
| Autistic Preoccupations | 12.7 (7.7) | 13.4 (8.1) | 15.9 (7.2) | 14.2 (6.9) | | 11.9 (5.9) | 12.9 (5.8) |
| **RBS-R subscales** | n=15 | n=15 | n=11 | n=14 | | n=14 | n=14 |
| Stereotypic Behavior | 2.6 (2.5) | 2.6 (2.9) | 2.9 (2.9) | 3.7 (2.9) | | 2.8 (3.2) | 3.6 (3.4) |
| Self-injurious Behavior | 1.0 (1.2) | 1.3 (2.1) | .9 (1.1) | 2.4 (5.6) | | 1.0 (1.8) | 1.4 (3.0) |
| Compulsive Behavior | 1.9 (2.4) | 2.1 (2.5) | 2.8 (4.5) | 1.8 (1.8) | | 1.2 (1.5) | 2.1 (2.8) |
| Ritualistic Behavior | 3.5 (2.8) | 3.9 (3.1) | 3.6 (3.7) | 3.0 (3.2) | | 1.5 (2.0) | 2.6 (2.5) |
| Sameness Behavior | 6.9 (4.6) | 5.2 (4.2) | 7.2 (7.0) | 4.9 (5.0) | | 3.2 (3.6) | 4.4 (4.4) |
| Restricted Interests | 1.9 (2.1) | 1.7 (1.8) | 1.9 (2.1) | 1.7 (1.6) | | .6 (.9) | 1.3 (1.7) |
| **SP-NL quadrants** | n=14 | n=14 | n=10 | n=15 | | n=15 | n=15 |
| Low Registration | 57.1 (9.5) | 60.4 (10.0) | 59.0 (11.6) | 52.5 (10.5) | | 58.5 (9.6) | 52.3 (8.7) |
| Sensation Seeking | 96.7 (16.1) | 102.5 (15.8) | 103.7 (19.2) | 97.8 (17.2) | | 108.7 (14.5) | 106.1 (16.9) |
| Sensory Sensitivity | 74.2 (10.4) | 77.2 (11.6) | 77.3 (11.9) | 71.7 (10.1) | | 77.8 (7.2) | 76.4 (9.8) |
| Sensation Avoiding | 100.6 (14.8) | 107.0 (15.7) | 103.4 (12.3) | 100.0 (15.3) | | 107.5 (15.7) | 103.4 (19.6) |
| **SP-SC quadrants** | n=12 | n=12 | n=7 | n=11 | | n=11 | n=10 |
| Low Registration | 57.4 (13.6) | 60.0 (9.2) | 54.9 (5.5) | 57.2 (12.5) | | 58.6 (10.9) | 57.6 (13.5) |
| Sensation Seeking | 35.6 (9.8) | 36.5 (8.4) | 36.1 (6.7) | 37.3 (9.6) | | 41.6 (9.9) | 40.7 (11.2) |
| Sensory Sensitivity | 49.4 (10.2) | 50.3 (7.9) | 47.6 (8.6) | 48.3 (6.1) | | 52.3 (9.5) | 49.8 (8.7) |
| Sensation Avoiding | 64.0 (15.7) | 65.4 (12.9) | 60.7 (10.0) | 60.8 (9.0) | | 63.7 (9.4) | 62.9 (11.9) |
| **ABC subscales** | n=15 | n=15 | n=11 | n=14 | | n=14 | n=14 |
| ABC Total | 55.3 (23.9) | 51.7 (22.8) | 42.6 (24.1) | 50.0 (26.9) | | 32.4 (16.0) | 35.1 (18.7) |
| Irritability | 17.1 (9.1) | 16.5 (8.6) | 13.2 (8.8) | 13.1 (10.3) | | 6.9 (4.3) | 7.9 (6.7) |
| Lethargy | 7.1 (8.7) | 5.5 (5.0) | 5.9 (4.3) | 9.6 (8.7) | | 7.0 (7.0) | 7.4 (8.1) |
| Stereotypic Behavior | 3.9 (5.2) | 3.6 (4.2) | 2.3 (2.1) | 5.8 (6.7) | | 2.9 (3.4) | 4.5 (5.3) |
| Hyperactivity | 23.7 (10.9) | 22.6 (11.1) | 16.8 (12.3) | 18.4 (8.2) | | 12.9 (6.3) | 13.1 (7.7) |
| Inappropriate Speech | 3.5 (2.4) | 3.5 (2.4) | 4.4 (3.4) | 3.2 (2.1) | | 2.6 (2.4) | 2.2 (1.8) |
| **BRIEF-parent subscales** | n=15 | n=15 | n=11 | n=15 | | n=15 | n=14 |
| Inhibit | 23.3 (4.6) | 23.2 (4.1) | 22.2 (5.6) | 21.8 (4.6) | | 19.5 (4.4) | 20.0 (4.5) |
| Shift | 17.9 (3.3) | 17.3 (3.4) | 18.9 (2.7) | 17.2 (3.4) | | 15.5 (3.6) | 16.4 (3.3) |
| Emotional Control | 23.8 (3.9) | 23.6 (4.9) | 23.4 (5.2) | 22.1 (5.5) | | 19.3 (4.2) | 19.6 (4.0) |
| Initiate | 18.5 (4.0) | 18.0 (4.3) | 18.6 (3.5) | 17.9 (3.3) | | 17.3 (3.6) | 17.1 (3.4) |
| Working Memory | 24.4 (4.3) | 23.9 (4.3) | 21.9 (4.4) | 24.1 (3.8) | | 22.7 (3.8) | 23.6 (3.7) |
| Plan/Organize | 23.2 (4.3) | 22.9 (4.7) | 22.7 (5.0) | 24.7 (3.7) | | 24.7 (4.8) | 25.0 (4.3) |
| Organization of Materials | 13.5 (3.5) | 14.1 (3.7) | 13.4 (3.9) | 12.7 (3.8) | | 12.8 (2.9) | 12.8 (2.9) |
| Monitor | 19.7 (3.4) | 19.3 (3.4) | 19.0 (3.6) | 18.9 (3.2) | | 18.9 (2.9) | 19.2 (3.2) |
| **BRIEF-teacher subscales** | n=13 | n=13 | n=8 | n=11 | | n=11 | n=9 |
| Inhibit | 23.5 (4.9) | 21.4 (5.7) | 22.4 (4.0) | 19.8 (5.1) | | 18.0 (6.5) | 19.6 (6.2) |
| Shift | 19.6 (5.7) | 19.3 (4.6) | 20.4 (4.5) | 21.3 (3.3) | | 20.9 (5.3) | 20.2 (4.6) |
| Emotional Control | 17.5 (5.3) | 17.9 (4.9) | 17.3 (4.6) | 17.3 (4.6) | | 16.3 (4.5) | 16.6 (4.1) |
| Initiate | 14.2 (3.8) | 13.4 (3.9) | 13.8 (3.3) | 14.7 (1.8) | | 15.2 (3.3) | 14.0 (3.4) |
| Working Memory | 20.4 (5.8) | 20.9 (5.9) | 21.6 (5.4) | 23.0 (2.1) | | 21.7 (2.8) | 20.8 (3.5) |
| Plan/Organize | 17.6 (4.6) | 18.0 (3.8) | 17.3 (4.1) | 19.1 (1.8) | | 19.4 (4.2) | 19.4 (4.2) |
| Organization of Materials | 12.6 (3.7) | 12.6 (4.3) | 13.4 (4.1) | 10.7 (2.6) | | 10.9 (3.2) | 10.8 (2.7) |
| Monitor | 23.3 (4.0) | 22.2 (4.8) | 23.0 (4.0) | 22.7 (4.1) | | 20.9 (5.6) | 20.6 (5.9) |
| **Note:** Data are mean (SD). ABC = Aberrant Behavior Checklist; BRIEF = Behavior Rating Inventory of Executive Function; RBS-R = Repetitive Behaviors Scale-Revised; SP-NL = Sensory Profile-2; SP-SC = Sensory Profile School Companion; SRS-2 = Social Responsiveness Scale-2. | | | | | | | |

# Supplementary table 3

| **Table S3.** Individual potassium levels split out per treatment group | | | | | | | | | | | | | | | | | | | | | | | | |
| --- | --- | --- | --- | --- | --- | --- | --- | --- | --- | --- | --- | --- | --- | --- | --- | --- | --- | --- | --- | --- | --- | --- | --- | --- |
| **BL** | | **D4** | | **D7** | **Extra^1^** | | **D14** | | | **Extra^2^** | | **Extra^3^** | | **D28** | **Extra^4^** | | **Extra^5^** | | | **Extra^6^** | | **D56** | **Extra^7^** | |
| **Bumetanide group** | | | | | | | | | | | | | | | | | | | | | | | | |
| 4.4 | 4.3 | | 4.2 | | |  | | 4.1 |  | |  | | 4.4 | | |  | |  |  | | **DR** | | |  |
| 3.5 | 4.3 | | 3.9 | | |  | | 4.0 |  | |  | | 3.8 | | |  | |  |  | | 3.4 | | | 3.6 |
| 4.3 | 4.1 | | 4.0 | | |  | | 3.5 | 3.9 | |  | | **DR** | | |  | |  |  | |  | | |  |
| 4.2 | 4.1 | | 4.0 | | |  | | 4.2 |  | |  | | 3.7 | | |  | |  |  | | 3.9 | | |  |
| 4.1 | 4.7 | | 4.2 | | |  | | 4.0 |  | |  | | 4.1 | | |  | |  |  | | 4.0 | | |  |
| 3.9 | 3.9 | | 4.3 | | |  | | 4.0 |  | |  | | 3.6 | | |  | |  |  | | 4.0 | | |  |
| 3.9 | 3.9 | | 4.1 | | |  | | 4.2 |  | |  | | 4.1 | | |  | |  |  | | 4.1 | | |  |
| 3.8 | 4.0 | | 4.1 | | |  | | 4.2 |  | |  | | 3.9 | | |  | |  |  | | 3.8 | | |  |
| 4.1 | 3.9 | | 3.4^b^ | | | 3.7 | | 3.3 |  | |  | | 3.5 | | |  | |  |  | | 3.6 | | |  |
| 4.2 | 3.9 | | 4.2 | | |  | | 3.9 |  | |  | | 4.0 | | |  | |  |  | | 3.8 | | |  |
| 3.9 | 4.1 | | 4.3 | | |  | | 3.9 |  | |  | | 3.9 | | |  | |  |  | | 3.6 | | |  |
| 4.1^a^ | 4.4 | | 3.6 | | |  | | 3.9 |  | |  | | 3.5 | | |  | |  |  | | 3.6 | | |  |
| 3.9 | 3.6 | | 3.6 | | |  | | 3.3 | 3.7 | |  | | 3.2 | | | 3.2 | | 3.8 | 3.5 | | 3.5 | | |  |
| 4.1 |  | | 3.7 | | |  | | 3.5 |  | |  | | 3.6 | | |  | |  |  | | 3.5 | | |  |
| 3.8 |  | | 3.5 | | |  | |  | 3.0 | | 3.9 | | 3.4 | | |  | |  |  | | 3.4 | | |  |
| 4,1 | 4.0 | | 3.9 | | |  | | 3.7 |  | |  | | 3.7 | | |  | |  |  | | 3.7 | | |  |
| 4.0 |  | | 5.0 | | |  | | 3.5 |  | |  | | 4.1 | | |  | |  |  | | 3.5 | | |  |
| 4.0 | 3.9 | | 3.7 | | |  | | 3.4 | 3.9 | |  | | 4.0 | | |  | |  |  | | 4.1 | | |  |
| 4.0 | 3.9 | | 3.8 | | |  | | 3.3 |  | |  | | **DR** | | |  | |  |  | |  | | |  |
| **Placebo group** | | | | | | | | | | | | | | | | | | | | | | | | |
| 4.1 | 4.3 | | 4.0 | | |  | | 4.1 |  | |  | | 4.0 | | |  | |  |  | | 4.4 | | | **DR** |
| 3.6 | 4.3 | | 4.0 | | |  | | 5.3 |  | |  | | 4.4 | | |  | |  |  | | 4.0 | | |  |
| 4.0 | 4.7 | | 4.5 | | |  | | 4.7 |  | |  | | 4.3 | | |  | |  |  | | 4.3 | | |  |
| 4.1 | 4.1 | | 4.2 | | |  | | 5.3 |  | |  | | 4.5 | | |  | |  |  | | 4.3 | | |  |
| 4.0 |  | | 3.8 | | |  | | 4.7 |  | |  | | **DR** | | |  | |  |  | |  | | |  |
| 4.2 | 3.9 | | 3.9 | | |  | | 4.1 |  | |  | | 4.0 | | |  | |  |  | | 3.8 | | |  |
| 4.0 | 4.3 | | 4.2 | | |  | | 3.9 |  | |  | | 4.1 | | |  | |  |  | | 4.2 | | |  |
| 4.4 | 4.7 | | 4.6 | | |  | | 4.0 |  | |  | | 4.4 | | |  | |  |  | | 4.2 | | |  |
| 3.6 | 3.7 | | 3.9 | | |  | | 3.8 |  | |  | | 3.9 | | |  | |  |  | | 3.9 | | |  |
| 4.3 |  | | 4.1 | | |  | | 4.0 |  | |  | | 5.1 | | |  | |  |  | | 5.4 | | |  |
| 4.3 |  | | 4.2 | | |  | | 4.6 |  | |  | | 4.3 | | |  | |  |  | | 3.7 | | |  |
| 4.3 |  | | 4.3 | | |  | | 3.8 |  | |  | | 4.1 | | |  | |  |  | | 4.2 | | |  |
| 4.1 |  | | 4.6 | | |  | | 4.7 |  | |  | | 5.5 | | |  | |  |  | | 4.1 | | |  |
| 3.8 | 4.3 | | 4.1 | | |  | | 4.0 |  | |  | | 3.9 | | |  | |  |  | | 4.1 | | |  |
| 3.9 | 4.2 | | 4.2 | | |  | | 3.9 |  | |  | | 3.9 | | |  | |  |  | | 4.0 | | |  |
| 3.9 | 4.5 | | 4.0 | | |  | | 4.0 |  | |  | | 4.2 | | |  | |  |  | | 4.5 | | |  |
| 3.8 |  | | 4.1 | | |  | | 4.3 |  | |  | | 4.5 | | |  | |  |  | | 4.3 | | |  |
| 4.5 |  | | 4.3 | | |  | | 4.0 |  | |  | | 4.5 | | |  | |  |  | | 4.1 | | |  |
| 4.0 |  | | 4.2 | | |  | | 4.2 |  | |  | | 4.2 | | |  | |  |  | | 4.5 | | |  |
| **Note:** Data are mmol/L; BL = baseline; DR = drop out. ^a^haemolytic sample ^b^visit at D10 | | | | | | | | | | | | | | | | | | | | | | | | |
